# Use of Leg-Mounted Monitors to Assess the Effects of Treponeme-Associated Hoof Disease on Elk (*Cervus canadensis*) Activity

**DOI:** 10.3390/ani16020306

**Published:** 2026-01-19

**Authors:** Trent O. Hill, Lisa A. Shipley, Steven N. Winter, Holly R. Drankhan, Kong Moua, Margaret A. Wild

**Affiliations:** 1 Department of Veterinary Microbiology and Pathology, College of Veterinary Medicine, Washington State University, Pullman, WA 99164, USA; trent.hill@wsu.edu (T.O.H.);; 2 School of the Environment, Washington State University, Pullman, WA 99164, USA; 3 Advanced Telemetry Systems, 470 First Ave. NW, Isanti, MN 55040, USA

**Keywords:** activity, elk, lameness detection, remote monitoring, treponeme-associated hoof disease

## Abstract

Treponeme-associated hoof disease (TAHD) is an emerging disease of free-ranging elk (*Cervus canadensis*) in the northwestern United States. Affected elk develop chronic foot lesions, lameness, debilitation, and an apparent increase in mortality, but the onset of lameness and associated changes in activity are not fully understood. We evaluated the accuracy of a newly developed leg-mounted tri-axial accelerometer monitor (Advanced Telemetry Systems) on captive elk and collected monitor-derived data to assess activity before and during an experimental TAHD challenge. Monitors provided reliable data with 85% overall accuracy of the continuous onboard classification of activity as standing, moving, or bedded against direct visual observation using seven healthy elk. Further, following TAHD challenge, monitor-derived data were able to detect that treatment elk exhibiting abnormal locomotion spent more time bedded and less time moving or standing. During the challenge period, treatment elk spent roughly 10% more of the day bedded than control elk. These findings suggest that leg-mounted activity monitors can detect changes in elk activity and may serve as a useful tool for future wildlife disease monitoring efforts.

## Simple Summary

Impacts from treponeme-associated hoof disease (TAHD) on individual elk and populations of free-ranging elk are of concern to interested publics and wildlife managers where the disease occurs in the northwestern United States. Lesions from TAHD range from minor skin ulcers on the feet to malformed and sloughed hoof capsules. Monitoring wild animals is challenging, and although lameness and debilitation may be observed in elk with severe lesions, the impacts of TAHD on elk activity throughout the disease course and in remote locations are difficult to determine. Remote monitoring of elk for changes in activity may provide a means for early detection and monitoring of impacts from TAHD. We evaluated a newly designed leg-mounted activity monitor that categorized activity as standing, moving, or bedded and remotely transmitted data to a receiver. We found that the monitor functioned reliably when placed on captive elk, accurately categorized their activity, and detected reductions in activity associated with mild TAHD lesions. Successful use in captive elk provides confidence for moving forward with further refinement of the system for deployment in free-ranging wildlife to evaluate impacts of TAHD and potentially other diseases.

## 1. Introduction

Treponeme-associated hoof disease (TAHD) is an emerging infectious disease affecting free-ranging elk (*Cervus canadensis*) in the northwestern U.S. [1,2]. TAHD is characterized by erosive or ulcerative pododermatitis that generally originates on the interdigital skin and can progress to include sole ulceration, undermining of the heel bulb, and broken, sloughed, or overgrown hoof capsules [1,3,4]. Severity of gross lesions is assessed using a four-point grading scale [3]. Infected elk often exhibit lameness that leads to debilitation and apparent increases in mortality [4,5]. TAHD-associated lameness may result in more time spent bedded and, thereby, limit key activities such as moving and foraging, as has been reported for cattle with lameness [6]. While these TAHD-related impacts are yet to be reported in free-ranging elk, reduced time foraging would be expected to result in loss of body condition, which may influence survival and fecundity, and increased likelihood of mortality from predation or trauma.

Remote monitoring could be useful for detecting changes in activity associated with development and progression of TAHD lesions and associated lameness for elk in the wild where direct visual observations of behavior are time-intensive and difficult to conduct at long distances and in remote settings [7]. For example, reduced activity could serve as an early indicator of disease that signals the need for increased monitoring. Additionally, assessing changes in activity over time could track impairment, mortality, or disease recovery in free-ranging elk. This information could contribute to our understanding of how the disease influences elk fitness, contributes to changes in vital rates (e.g., birth rate, death rate), and alters population trends.

Remote monitoring of activity has been used to study a variety of behaviors in wildlife [8,9,10]. Tracking devices equipped with tip-switches or global positioning systems (GPS) have been used to coarsely classify behavioral states as active and non-active in elk [11], bears (*Ursus thibetanus japonicus*; [12]), and migrating birds [8]. Collars and eartags with activity sensors that measure changes in body posture, acceleration, and angles associated with various activities that have been used in several wild species, including elk, provide finer scale activity classification but require extensive validation and/or post-processing on large volumes of raw data [9,10,13,14,15]. Activity monitors designed for use in humans have also been evaluated in wildlife but lack the capability to remotely transmit data [16]. As such, currently available wildlife monitors are insufficient to detect the fine-scale changes in activity (i.e., time spent bedded, standing, and moving) and remotely transmit real-time classification of activity necessary for feasibly studying TAHD in free-ranging elk.

Intensive activity monitoring systems used to monitor livestock, particularly dairy cattle, are evolving from experimental evaluations to commercial farm-scale applications for monitoring health, detecting lameness, and assessing productivity [17,18,19,20,21]. Monitors used on livestock often employ collar-, ear-, and/or leg-mounted accelerometers to identify bedded, feeding, ruminating, standing, and moving activities [22,23,24]. Leg-mounted tri-axial accelerometers on cattle provide reliable data, particularly for standing and bedded activities [21,25], and have been used to detect early signs of lameness from hoof disorders [26,27]. Additionally, in contrast to most activity monitors available for use in wildlife, contemporary livestock activity monitors incorporate onboard classification of defined activities, reducing the need for extensive post-processing of raw data [21,28,29].

Intensive monitoring technologies used in livestock could be a valuable tool for studying wild ungulates by enabling continuous, remote activity monitoring. Specifically, leg-mounted activity monitors have the potential to enhance detection and study effects of disease-associated activity changes, particularly for chronic diseases such as TAHD. Therefore, we developed a leg-mounted activity monitor designed to classify elk activity into three categories (moving, standing, and bedded) using an onboard classification system and evaluated accuracy of the monitors on captive elk. Following validation, we used monitor-derived data to assess changes in activity before and during an experimental TAHD challenge study. We hypothesized that the activity of elk in both the treatment and control groups would be similar during the pre-challenge period but that following experimental TAHD challenge, the activity of treatment elk would decrease, represented as more time spent bedded. Findings provide insights into changes in activity of captive elk challenged with TAHD and suggest leg-mounted monitors may be a useful tool to detect and assess impacts of disease in free-ranging elk.

## 2. Materials and Methods

This study was conducted opportunistically in conjunction with an experiment aimed at developing a model to produce rapid reproduction of TAHD lesions in captive elk [30]. All procedures were approved by the Washington State University (WSU) Institutional Animal Care and Use Committee. We used seven adult female Rocky Mountain elk originally captured from the Starkey Experimental Forest and Range, Oregon, USA, at approximately 9 months of age, and maintained at the WSU Elk Research Facility in Pullman, Washington, USA. Elk were housed individually in outdoor pens (4.9 m × 6.1 m) with concrete floors and wood-shaving bedding during September to November 2024. We provided elk with a maintenance diet consisting of daily feedings of grass hay supplemented with a specially formulated pelleted feed (Mazuri Exotic Animal Nutrition, St. Louis, MO, USA) and ad libitum access to water and mineral salt blocks.

**Figure 1:**
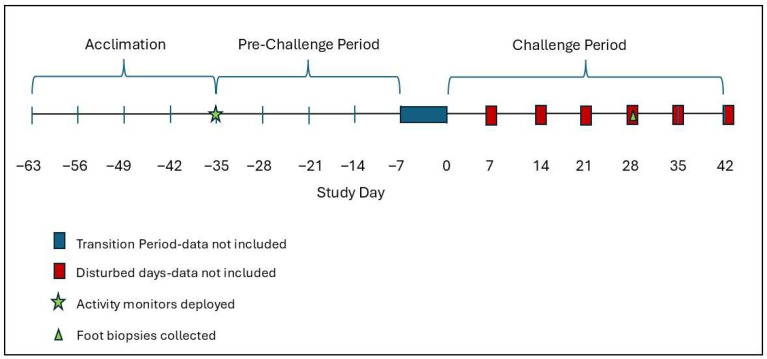
Timeline of study evaluating activity monitors in captive elk (*Cervus canadensis*) that included an acclimation period prior to study initiation, a 4-week pre-challenge period, a transition period when data were not included in activity analyses, and a 6-week challenge period. Elk were handled at weekly intervals during the challenge period and data from the day of handling and previous day are not included in activity analyses.

**Figure 2:**
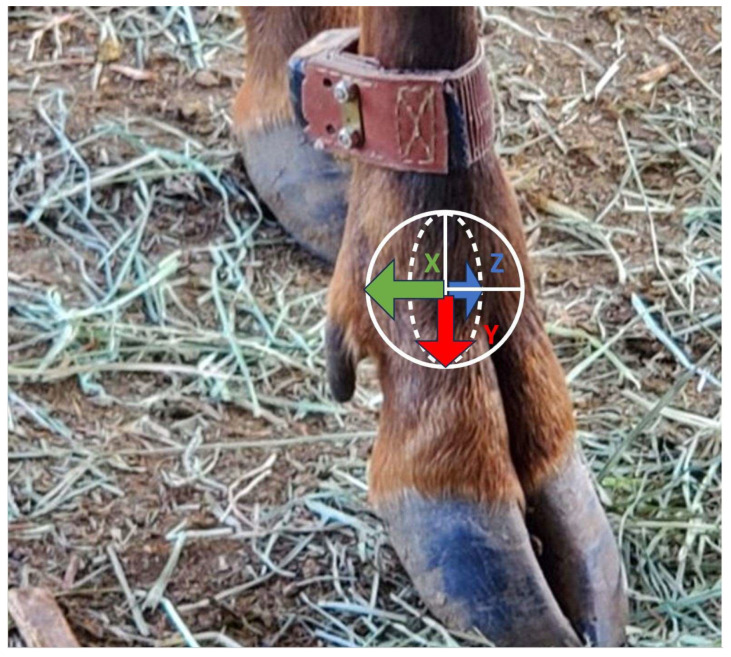
Advanced Telemetry Systems tri-axial (x, y, and z axes, as illustrated) activity monitor attached to the lower metacarpus of a captive elk (*Cervus canadensis*).

We assigned each elk to either a treatment group (*n* = 5) or a control group (*n* = 2). Our study design consisted of a 4-week acclimation and 4-week pre-challenge monitoring period followed by a 1-week transition period when elk were handled, sampled, and initially fitted with foot wraps, and then a 6-week challenge period (Figure 1). During the challenge period (Day 0–42 or 44), treatment elk were topically exposed to an inoculum of infectious material to induce TAHD, whereas control elk received a mock inoculum using the same handling procedures [30]. Elk were immobilized for study procedures using a combination of butorphanol, azaperone, and medetomidine (BAM; Wedgewood Pharmacy, Swedesboro, NJ, USA) following a fasting period of 24–36 h to avoid complications during sedation. The first immobilization event occurred one day prior to the pre-challenge period. At this time, elk were fitted with an activity monitor on the front right lower limb (Figure 2). At weekly immobilizations during the challenge period, the activity monitor was inspected for damage, the leg was checked for any monitor-associated trauma, and the hind feet were examined, sampled, treated with inoculum, and rewrapped in accordance with the TAHD challenge protocol [30]. Additionally, on challenge day 28, local anesthetic was injected, and a 5 mm punch biopsy was aseptically collected from the interdigital space of both hind feet of all elk to evaluate tissues for TAHD [30]. An analgesic (Meloxicam, 1 mg/kg) was administered orally to all elk on challenge day 28 and to any elk that exhibited a locomotion score ≥ 2. Lesion severity was visually scored by a veterinarian at three time points (challenge days 14, 28, and 42 or 44) using a previously reported four-point grading system [3,30]. The study concluded after all treatment elk developed hoof lesions consistent with TAHD.

Activity data were continuously recorded using leg-mounted activity monitors during the study. Monitors were designed and produced by Advanced Telemetry Systems (Isanti, MN, USA) to automatically classify activity using onboard accelerometer data. Purchase cost per monitor was USD 680. The monitors consisted of a tri-axial accelerometer, a microcontroller, and a radio frequency communication module, along with a battery. The monitor was attached to a 4 cm wide leather band with an expandable section (total weight 125 g) and fitted with flush mount bolts and lock nuts (Figure 2). Monitors classified the time (in seconds) that an animal spent performing three specific activities: moving, standing, and bedded. The three axes (Figure 2) of the accelerometer detected movement from the rate of change in the axes. If the rate of change exceeded a predetermined movement threshold, it assigned that time period as moving. Otherwise, the monitor examined the actual reading of the axis parallel to the leg. An accelerometer at rest with gravitational force applied only in one axis direction would report a magnitude of 1 *g* (i.e., acceleration due to gravity that is approximately 9.8 m/s^2^ on Earth). In contrast, if an axis was perpendicular to the downward force of gravity, the value reported would be 0 *g* in that axis direction. When strapped to an animal, one axis of the accelerometer was essentially parallel to the leg (i.e., leg axis). In the monitor, when the leg axis was vertical (as in a standing animal), reported accelerometer values would be closer to 1 *g* in magnitude. When the leg axis was horizontal (as in a bedded animal), the reported value would be closer to 0 *g*. Because ideal accelerometer orientation was not assumed due to variation in manufacturing, attachment, and individual animals, the monitor used a cutoff at 45° from vertical for classifying the standing activity and 55° from vertical for classifying bedded activity. All activity status data were recorded by the microcontroller on the monitor and offloaded via a radio frequency communication interface with a Wildlink W100 module (Advanced Telemetry Systems, Isanti, MN, USA) located within 13 m of the monitored elk; however, we also evaluated the ability to receive data line-of-sight up to 122 m, which was the farthest distance possible in the research facility. The Wildlink module was used to program the recording intervals for monitors, which for this study were set to assign time (in seconds) spent moving, standing, and bedded in each 1 min interval to acquire fine-scale data for validating monitors and assessing activity.

To assess the accuracy of monitors, we compared the classifications of data from the monitors with observed activities collected using a focal sampling technique during the pre-challenge period [31]. One trained technician conducted six (two morning, two midday, and two late afternoon) 10 min focal sampling sessions per individual elk across 2 weeks. The activities of interest (i.e., moving, standing, bedded) were recorded on a Surface Pro 9 laptop (Microsoft, Redmond, WA, USA) using BORIS (Behavioral Observation Research Interactive Software) software (version 8.27.8) [32]. Focal sampling session data, recorded in seconds by BORIS, were converted to minutes and synchronized in time to coincide with activity monitor output. We compared the number of minutes in which monitors classified each activity against our reference visual observations using a confusion matrix to determine monitor accuracy for each activity category and overall accuracy [33].

Additionally, we explored how monitor-derived activity differed as elk developed early signs of TAHD-associated lameness. During both the pre-challenge and challenge periods, five trained observers visually assessed each elk for 2–3 min daily and assigned a locomotion score based on previously reported criteria: 0 indicated the elk was sound, 1 indicated an imperfect gait, 2 indicated lameness, and 3 indicated severe lameness [4]. We compared the daily locomotion scores with activity by calculating the mean proportion of the day that elk spent engaged in each activity and 95% confidence intervals (CI) for means to illustrate differences.

We examined changes in activity associated with TAHD challenge and disease development by calculating the proportion of the day elk spent bedded and, within active time (standing or moving), the proportion of time spent moving based on monitor-derived data during the pre-challenge and challenge periods. In our preliminary visualization of data, we observed that activity differed on days elk were fasted as well as days when immobilized for handling. Therefore, we analyzed data from only the five contiguous “undisturbed” days per week during the challenge period. Similarly, we did not analyze data from the transition period (week 5) when elk were fasted or handled on most days of the week (Figure 1).

We modeled separately the proportion of the day that elk spent bedded and the proportion of active time spent moving using generalized linear mixed models with a beta distribution (hereafter ‘beta GLMM’) built using data from either the pre-challenge or challenge periods. We examined how variation in activity was associated with fixed effects of the group (control or treatment), time as Julian day, and additive and interactive effects between group and time. Elk identifiers were included as a random effect to control for repeated measures and different baselines of activity specific to individuals. Beta GLMMs were built with a logit link using the glmmTMB package (version 1.1.12 [34]) in R [35]. We ranked and evaluated support for models containing covariates and a model of no effect (i.e., a null model) using Akaike’s information criterion with a correction for small sample sizes (AICc) [36] with the MuMIn package (version 1.48.11 [37]).

## 3. Results

### 3.1. Reliability of Activity Monitors

The leg-mounted activity monitors functioned reliably throughout the duration of the study. All units remained attached firmly to the elk with no observed movement on the metacarpus that may have affected reliability of data and no signs of leg trauma, irritation, or injury from the monitor. Additionally, the data collection process was uninterrupted, with no instances of missed or corrupted data across study periods. Although data were collected at a distance of ≤13 m for this study, we successfully received transmitted data up to 122 m, the maximum line-of-sight distance tested.

### 3.2. Comparison of Monitor-Derived Data Against Visual Observations

Sixty minutes of observational data were successfully collected from each of the seven elk and used to determine monitor accuracy during the pre-challenge period. Of the 420 min observed, elk spent the majority of time standing (55%), followed by moving (39%), and bedded (6%). The monitors had an overall classification accuracy of 85%, with bedded (92%) and standing (88%) showing the highest agreement between visual observation and monitor output, while moving was correctly classified 78% of the time (Table 1).

**Figure 3:**
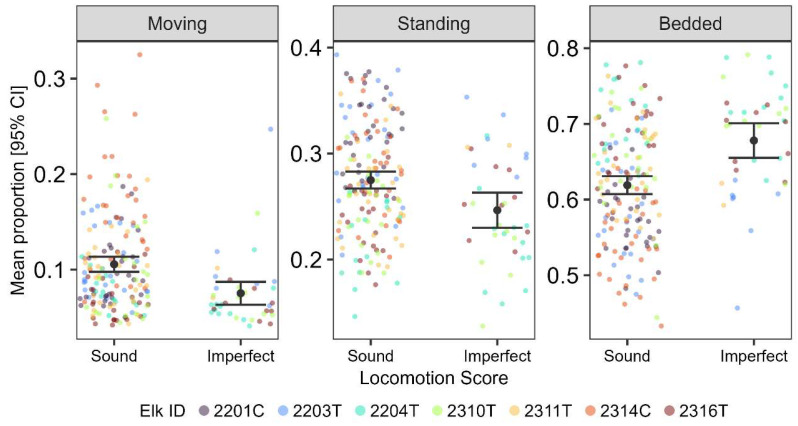
Mean proportion of the day that captive elk (*Cervus canadensis*) spent in each activity category (moving, standing, and bedded) and their corresponding locomotion score (sound or imperfect gait). Error bars represent 95% confidence intervals of the mean. Colored dots represent raw data points for individual elk (jittered so all are visible). Note the *y*-axis for each panel is different to improve visualizations.

Additionally, monitor-derived data reflected changes observed in locomotion scores. We observed that decreased daily activity generally corresponded with higher observed locomotion scores (Figure 3). On days when elk were scored as having imperfect gait (locomotion score 1), they spent smaller proportions of the day moving and standing and a larger proportion bedded compared to days when locomotion was scored as sound (locomotion score 0). One treatment elk (23-04) was observed to be lame (locomotion score 2) during three observational sessions in the challenge period and received oral Meloxicam on challenge days 36–42. Due to the small number of observations of locomotion score 2, these data were omitted from the analysis. Severe lameness (locomotion score 3) was never observed.

### 3.3. Changes in Activity

#### 3.3.1. Activity in the Pre-Challenge Period

**Figure 4:**
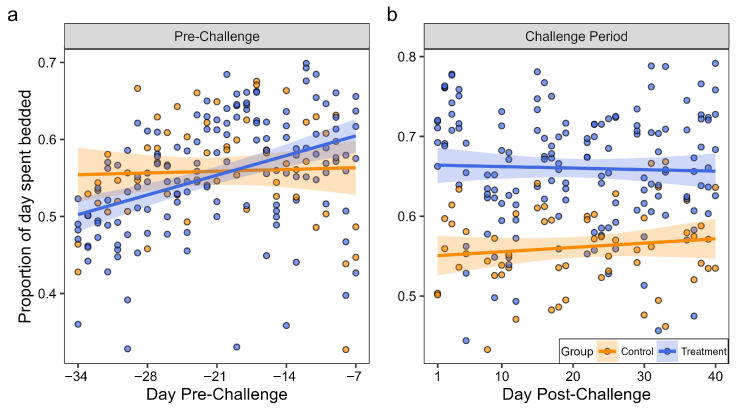
Trends in bedded activity in treatment and control captive elk (*Cervus canadensis*) wearing leg-mounted activity monitors during (**a**) pre-challenge and (**b**) challenge periods of a treponeme-associated hoof disease transmission study.

Our best-supported model explaining variation in bedded activity included the effects of group, day, and their interaction (Akaike weight, *w_i_* = 0.84; Table 2). We found a small, positive effect for the interaction between group and day (*β_group:day_* = 0.014, 95% CI: 0.004–0.023), suggesting that, contrary to our hypothesis, treatment and control elk exhibited different bedded activity over time during the pre-challenge period after accounting for differences among individuals. Relative to control animals, treatment elk exhibited a small increase over time in the proportion of the day spent bedded (1.4% increase each day; Figure 4a). In contrast, we did not find support for models assessing how the proportion of active time spent moving was associated with effects of group, day, or any combined effects in the pre-challenge period, based on the model of no effect receiving similar support to those with covariates (Table S1).

#### 3.3.2. Activity in the Challenge Period

After challenge, treatment elk developed minor TAHD lesions (grades I–II) whereas control elk showed no signs of disease [30]. The model containing only the effect of the group received the greatest probability of being the best model of the set (*w_i_* = 0.57; Table 2), suggesting that variation in bedded behavior was best explained by the variable describing whether elk belonged to the treatment or control group. As hypothesized, we found that following experimental challenge, treatment elk spent 10% more time bedded on average than control elk (Figure 4b). We found no support for the effect of time on bedded activity in the challenge period, meaning reductions in activity did not differ over time from the point of initial TAHD exposure and as TAHD lesions developed. Furthermore, as in the pre-challenge period, models assessing the proportion of active time spent moving received similar support to the model of no effect (Table S1).

## 4. Discussion

The demonstrated accuracy and reliability of the newly developed leg-mounted activity monitors equipped with onboard classification technology indicate that they are a useful tool for monitoring activity in elk. The high level of agreement between visual observations and automatic onboard activity classification of standing (88%) and bedded (92%) activity confirmed that pre-set cutoffs for classifying these activities (45° from vertical for standing and 55° from vertical for bedded) provided reliable ranges for detection of leg orientation by the monitor. The benefit of real-time classification was offset by a lower accuracy than the ≥98% reported for experimental leg-mounted monitors that required post-processing of data used in cattle [25] or prior data training of the classification system used in rhinoceros (*Ceratotherium simum* and *Diceros bicornis*) [38]. While acceptable, moving activity by elk had a somewhat lower accuracy (78%) due to occasionally being misclassified as standing or bedded. The less-accurate classification of moving activity in our experiment may have been related to altered movement in the physical constraints of the captive environment. Elk confined to relatively small pens frequently turned, shifted, or exhibited brief bouts of movement, which may have produced less consistent acceleration patterns compared to the more unidirectional movement expected in free-ranging individuals. These fragmented motion patterns may have challenged the threshold-based classifiers and acceleration sensors.

The ability of monitors to continuously classify activity extends the capacity for researchers to gather fine-scale activity data over long periods. By eliminating the need for the post-processing of raw accelerometer data, the onboard classification system simplifies data workflow and facilitates timely interpretation, potentially enabling real-time health assessment of wild or captive animals in future applications [39,40]. Further, the monitors allow data to be collected over a 24 h period, capturing activity when visual observations would not be possible. This benefit would be especially valuable in free-ranging wildlife, where visual observations are time-intensive and difficult to conduct at long distances and in remote settings. The ability to remotely monitor animal activity is advantageous in both free-ranging and captive settings by reducing disturbance because data can be gathered without the need for direct human presence. This observation is supported by the difference in the proportion of time elk spent bedded during visual observations (6%) versus continuous monitoring (on average > 50%; Figure 4a). Finally, the decrease in daily activity observed in association with higher locomotion score (Figure 3) further confirmed that monitors can reliably detect changes in activity over time.

Captive elk experimentally inoculated with TAHD provided a valuable opportunity to examine changes in activity using the newly developed activity monitors. During the pre-challenge period, differences in activity based on treatment group alone were not supported by our models. However, differences were observed over time during the pre-challenge period; monitor data demonstrated that treatment elk exhibited a small daily increase (1.4% per day) in time spent bedded but not a concurrent decrease in the proportion of active time spent moving prior to challenge initiation. The observed daily increase in time bedded through the pre-challenge period may reflect ongoing acclimation to housing in individual pens that occurred over time despite allowing 4 weeks of housing before the start of the pre-challenge monitoring period. These findings reflect the importance of allotting sufficient time for acclimation to new environments and suggest that informal visual observations of behavior may not accurately reflect acclimation to new environments.

After challenge, treatment elk, all of which developed minor TAHD lesions (grades I–II; [30]), spent 10% more time bedded on average than control elk that showed no signs of disease. While this difference in activity between the treatment and control groups supported our hypothesis and was observed concurrent with TAHD lesions, it is unclear why the difference was apparent even on the first day of the challenge period (Figure 4b). Regardless, the observed change in activity suggests that using monitor data provides a sensitive and effective way to detect subtle alterations in activity, including those that are associated with disease. Studies using remote monitoring of livestock were able to identify the onset of lameness and detect a change in activity as lameness developed, in part because lame cows spent more time bedded [26,41]. We did not detect an effect of time with respect to reductions in activity from initial TAHD exposure and as TAHD lesions developed. However, elk in our study exhibited only mild lesions and alterations to locomotion, contrary to a previous TAHD transmission study in which moderately severe (grade III) lesions were induced and locomotion scores of 2 or 3 were common [4]. Although all treatment elk developed TAHD, lesion severity was limited to grades I–II and many lesions were reduced or resolved by study termination [30]. Further, visually observed alterations in locomotion were minor, with 4 of 5 treatment elk reaching a maximum locomotion score of 1, and just three observations of score 2 in one elk. Induction of only mild lesions that were limited to the interdigital skin prevented us from assessing lameness and activity changes that we hypothesized would be associated with more severe lesions that involve the sole and heel bulb or impact hoof growth. Additionally, the use of wraps throughout the challenge period to facilitate rapid lesion progression likely acted like a padded bandage that reduced pressure and trauma to the foot. Because this study was designed to reduce animal pain and discomfort, it was not ideal for monitor evaluation. Thus, even though the increased bedding observed in treatment animals suggests monitor sensitivity to early changes in activity, the restricted range of lameness and lesion severity limited our ability to fully evaluate the monitors’ ability to detect impacts associated with TAHD.

While the captive setting allowed visual observations and evaluation of monitors under controlled conditions with a TAHD challenge study, the artificial environment may have obscured some changes in activity that would be expected in the wild. In addition to frequent changes in movement patterns associated with the small pens challenging the monitor’s ability to accurately classify moving activity, natural behaviors, such as foraging and long-distance movement, could not be evaluated in the captive setting. Although changes in the proportion of active time spent moving vs. active time spent standing were not detected in experimental elk, we investigated this difference because TAHD lesions and associated lameness would be expected to have a greater effect on moving behavior of wild elk. This reduction in movement could have significant impacts on elk well-being and survival. Free-ranging elk spend a greater proportion of their time moving while foraging, seeking shelter from weather, and avoiding predators [11,42,43]. For example, free-ranging elk spent 8–11% of their day traveling and 31–37% of the day feeding, which consisted of stop and go movements, and bedded or rested for 36–55% of the day [44,45]. In contrast, during the pre-challenge and challenge periods, captive elk in our study moved for just 9–14% of the day on average and bedded for 50% to over 65% of the day.

## 5. Conclusions

Our findings support the use of the newly developed leg-mounted activity monitors in future studies of activity in captive elk. The monitors functioned reliably, accurately categorized activity, and detected reductions in activity associated with mild TAHD lesions. Monitors were commercially available and reasonably priced, particularly given the simplified data collection that onboard activity classification provided. Further, our findings suggest the potential for successful use in free-ranging elk. Before deploying the monitors on free-ranging elk, however, modifications are required to extend the range of data collection. Our informal evaluation revealed line-of-sight transmission distances up to 122 m, although other studies using collar accelerometers have reported upload distances up to 800 m [8]. Modifications to the system, such as integrating higher-powered antennas or linking leg monitors to collars to boost data transmission, would extend functionality in field settings. When paired with traditional GPS collars, monitoring changes in activity over time could provide insights into the impacts of disease progression and its influences on vital rates, as well as broader-scale use of the monitors for activity surveillance to determine energy budgets, foraging activity, and habitat use in free-ranging ungulates.

## Figures and Tables

**Table 1:** Confusion matrix comparing activity classifications from leg-mounted activity monitors to visual observations in healthy captive elk (*Cervus canadensis*). Values indicate the number of 1 min intervals classified into each activity category (moving, standing, bedded).

		Actual Observation				
	Activity Type	Moving	Standing	Bedded	Total	Accuracy
Monitor Output	Moving	134	30	2	171	78%
	Standing	20	198	1	224	88%
	Bedded	1	1	23	25	92%
	Total	165	229	26	420	Overall: 85%

**Table 2:** Model selection results for the proportion of the day that elk (*Cervus canadensis*) spent bedded in pre-challenge and challenge periods of treponeme-associated hoof disease study.

	Model Notation ^1^	K	LL	AICc	ΔAICc	*w_i_*
Pre-Challenge	Bedded~Group × Day	6	270.13	−527.83	0.00	0.84
	Bedded~Day	4	266.03	−523.85	3.98	0.12
	Bedded~Group + Day	5	266.05	−521.79	6.04	0.04
	Bedded~1 (Model of no effect)	3	253.83	−501.53	26.30	0.00
	Bedded~Group	4	253.84	−499.49	28.34	0.00
Challenge	Bedded~Group	4	299.79	−591.38	0.00	0.57
	Bedded~Group + Day	5	299.79	−589.28	2.10	0.20
	Bedded~Group × Day	6	300.36	−588.30	3.07	0.12
	Bedded~1 (Model of no effect)	3	296.81	−587.51	3.87	0.08
	Bedded~Day	4	296.81	−585.43	5.94	0.03

Abbreviations: K, numbers of parameters; LL, log-likelihood; AICc, Akaike’s Information Criterion corrected for small sample size; *w_i_*, Akaike weight. ^1^ All models also contained elk ID as a random effect.

## Data Availability

The original data presented in the study are openly available in Hill, T.O.; Shipley, L; Winter, S.N.; Wild, M.A. Dataset: Use of leg-mounted monitors to assess the effects of treponeme-associated hoof disease on elk (*Cervus canadensis*) activity. Figshare. 2025 at https://doi.org/10.6084/m9.figshare.30795251 (accessed on 14 January 2026).

## Supplementary Materials

The following supporting information can be downloaded at: https://www.mdpi.com/article/10.3390/ani16020306/s1, Table S1: Model selection results evaluating effects of group (treatment or control), day, and their interaction on the proportion of active time that captive elk (*Cervus canadensis*) wearing leg-mounted activity monitors spent moving during pre-challenge and challenge periods of a treponeme-associated hoof disease transmission study.

## Abbreviations

The following abbreviations are used in this manuscript:

TAHD	Treponeme-associated hoof disease
GPS	Global positioning system
WSU	Washington State University
BORIS	Behavioral Observation Research Interactive Software
CI	Confidence interval
GLMM	Generalized linear mixed models
AICc	Akaike’s information criterion with a correction for small sample sizes
*w_i_*	Akaike weight
