# Supplementary material for: Use of Leg-Mounted Monitors to Assess the Effects of Treponeme-Associated Hoof Disease on Elk (Cervus canadensis) Activity
Source: Animals (Basel). 2026 Jan 19;16(2):306. doi: 10.3390/ani16020306 (PMC12837264; doi:10.3390/ani16020306)
Supplement: Supplementary file 1 [file animals-16-00306-s001.zip › animals-4053168-supplementary.pdf]

## Supplementary Materials
## Hill et al.

**Table S1.** Model selection results evaluating effects of group (treatment or control), day, and their interaction on the proportion of active time that captive elk (*Cervus canadensis*) wearing leg-mounted activity monitors spent moving during pre-challenge and challenge periods of a treponeme-associated hoof disease transmission study.

| Period | Model notation[1] | K | LL | AICc | ΔAICc | $w_i$ |
|---|---|---|---|---|---|---|
| Pre-Challenge | Moving ~ Group | 4 | 147.14 | -286.07 | 0.00 | 0.33 |
| | Moving ~ 1 (Model of no effect) | 3 | 146.05 | -285.97 | 0.10 | 0.31 |
| | Moving ~ Group × Day | 6 | 148.26 | -284.10 | 1.97 | 0.12 |
| | Moving ~ Group + Day | 5 | 147.16 | -284.02 | 2.05 | 0.12 |
| | Moving ~ Day | 4 | 146.08 | -283.95 | 2.12 | 0.11 |
| Challenge | Moving ~ 1 (Model of no effect) | 3 | 244.24 | -482.36 | 0.00 | 0.34 |
| | Moving ~ Group | 4 | 245.15 | -482.11 | 0.25 | 0.31 |
| | Moving ~ Day | 4 | 244.51 | -480.83 | 1.53 | 0.16 |
| | Moving ~ Group + Day | 5 | 245.43 | -480.56 | 1.80 | 0.14 |
| | Moving ~ Group × Day | 6 | 245.44 | -478.46 | 3.90 | 0.05 |

Abbreviations: K, numbers of parameters; LL, log-likelihood; AICc, Akaike's Information Criterion corrected for small sample size; $w_i$, Akaike weight. [1] All models also contained elk ID as a random effect.
